# Quantifying the differential relationship between red blood cell docosahexaenoic acid and cognition based on *APOE-e4* carriership in multiple cohorts: illustration of an emerging statistical approach

**DOI:** 10.1016/j.plefa.2025.102713

**Published:** 2025-10-25

**Authors:** Nathan A. Ryder, Jason Westra, Aleix Sala-Vila, Jack Wolf, William S. Harris, Nathan L. Tintle

**Affiliations:** a Fatty Acid Research Institute, Sioux Falls, SD, USA; b Hospital del Mar Research Institute, Barcelona, Spain; c Centro de Investigación Biomédica en Red de Fisiopatología de la Obesidad y Nutrición (CIBEROBN), Instituto de Salud Carlos III, Madrid, Spain; d University of Pennsylvania, Perelman School of Medicine, Philadelphia, PA, USA; e University of South Dakota, School of Medicine, Sioux Falls, SD, USA; f University of Illinois – Chicago, College of Nursing, Chicago, IL, USA

**Keywords:** DHA, Cognition, Aging, Harmonization, Data-sharing, Summary statistics

## Abstract

Evidence is mounting that red blood cell membrane levels of docosahexaeonic acid (DHA) are directly associated with improved cognitive performance, with emerging questions about whether this relationship may be stronger in some individuals vs. others, especially those at high genetic risk of cognitive impairment via the *APOLIPO-PROTEIN-E* (*APOE*) *ε4* allele. We investigated this relationship in *n* = 2158 participants without dementia from four separate cohorts (Offspring, Gen3, Omni I and Omni II) of the multi-cohort Framingham Heart Study, aged 50 to 70 years old. To demonstrate an emerging alternative of improved data sharing, we conducted the analysis using only cohort-level, summary data which is non-individually identifiable and easily shareable. We detected a statistically significant interaction between *APOE-ε4* status and DHA in multivariable models adjusting for relevant demographic and medical history covariates and predicting cognitive impairment (*p* = 0.039). Stratified adjusted model results detected a stronger inverse association between cognitive impairment and DHA for *APOE-ε4* carriers (−0.13; 95 % CI: (−0.20, −0.06) minutes per 1 % increase in DHA), as compared to non-carriers (−0.06; 95 % CI: (−0.11, −0.02)), though both were statistically significant (*p* < 0.05). We also analyzed the data using two, standard, widely used statistical approaches (1) Individual level data analysis (identical results) and (2) standard meta-analysis (slightly weaker evidence of interaction; *p* = 0.047). Our analysis demonstrates not only the putative importance of DHA on cognition, especially among *APOE-ε4* carriers, but the potential for an emerging class of data-sharing methods to accelerate the future pace of cross-cohort analyses by not requiring access to individual-level data.

## 1. Background

Alzheimer’s disease and related dementias (AD/ADRD) impose a substantial socioeconomic burden, enhanced by a lack of cost-effective pharmacologic approaches that slow AD/ADRD progression [22]. As a result, preventive strategies remain of paramount importance. Notably, evidence is emerging about the relationship between diet at mid-life and incident AD/ADRD [11], with particular interest in the role of the omega-3 fatty acid docosahexaenoic acid (DHA) [4], a major component of brain membranes [15]. Some prospective studies have examined the association between dietary DHA and incident AD/ADRD. A recent meta-analysis and systematic review of prospective cohort studies reported a lower risk for many (but not all) studies [23]. Such discrepancy could be explained, at least in part, by the limited use of blood DHA levels instead of highly variable and biased self-reported dietary self-report [13].

Recent work by our group on a single cohort has shown that blood DHA levels may be more strongly associated with a lower AD/ADRD risk among individuals carrying at least one *APOLIPOPROTEIN-E* (*APOE*) *ε4* allele [19], who are at higher risk of AD/ADRD [8]. It has been suggested that greater DHA intake in this population segment would help preserve cognitive performance before the onset of clinical symptomatology [7], but several studies examining the association between DHA and cognitive outcomes have reported divergent effects based on *APOE* genotypes [26]. However, there is still a dearth of multi-cohort evidence, that could provide a stronger message about the relationship between DHA and *APOE-ε4* in cognition. Meta-analysis of published results can be helpful in that purpose. However, standard meta-analyses are subject to publication bias and limited in that they cannot precisely account for analytic heterogeneity (e.g., different phenotype definitions; different covariate sets) among other known limitations (e.g., lack of statistical efficiency). However, to conduct *de novo*, multi-cohort analyses researchers typically must have individual level data access to multiple cohorts (directly or indirectly – via a collaborator). Importantly, there is a need for methodological innovation to minimize computational complexity and respect data privacy concerns while maximizing data access and utility. At the forefront of these innovations are computational methods that leverage pre-computed, non--individually identifiable summary statistics on cohort data to maximize functional understanding and clinical utility. One emerging set of pre-computed summary statistics (PCSS) are point estimates and standard errors from regression models of a disease or biomarker on a single predictor sometimes with limited covariate adjustment (e.g., Age, Sex and Principal Components (PCs)), supplemented with other PCSS [10]. While promising, a key concern of any set of PCSS is limitations in the possible downstream uses of such statistics. Recently, we have developed PCSS methods available in a published an R software package (pcsstools) which allow for (a) de novo covariate adjustment, and (b) different disease/biomarker definitions using only PCSS – not requiring access to individual level data [24,25,27].

Here, we apply these emerging methodologies to a four-cohort analysis of cognitive outcomes data with blood DHA levels to demonstrate the method’s feasibility on an emerging, meaningful scientific research question: Do blood DHA levels differentially associate with cognition by *APOE-ε4* status? We also will demonstrate that a straightforward extension of the method provides identical results to what is obtained if the analysis is conducted on the individual level data directly (individual participant data; IPD), and how the identical PCSS and IPD methods can, in this case, yield more power than standard meta-analysis.

## 2. Methods

### 2.1. Emerging PCSS method to conduct IPD-equivalent analyses using only cohort summary statistics

**Fig. 1.**
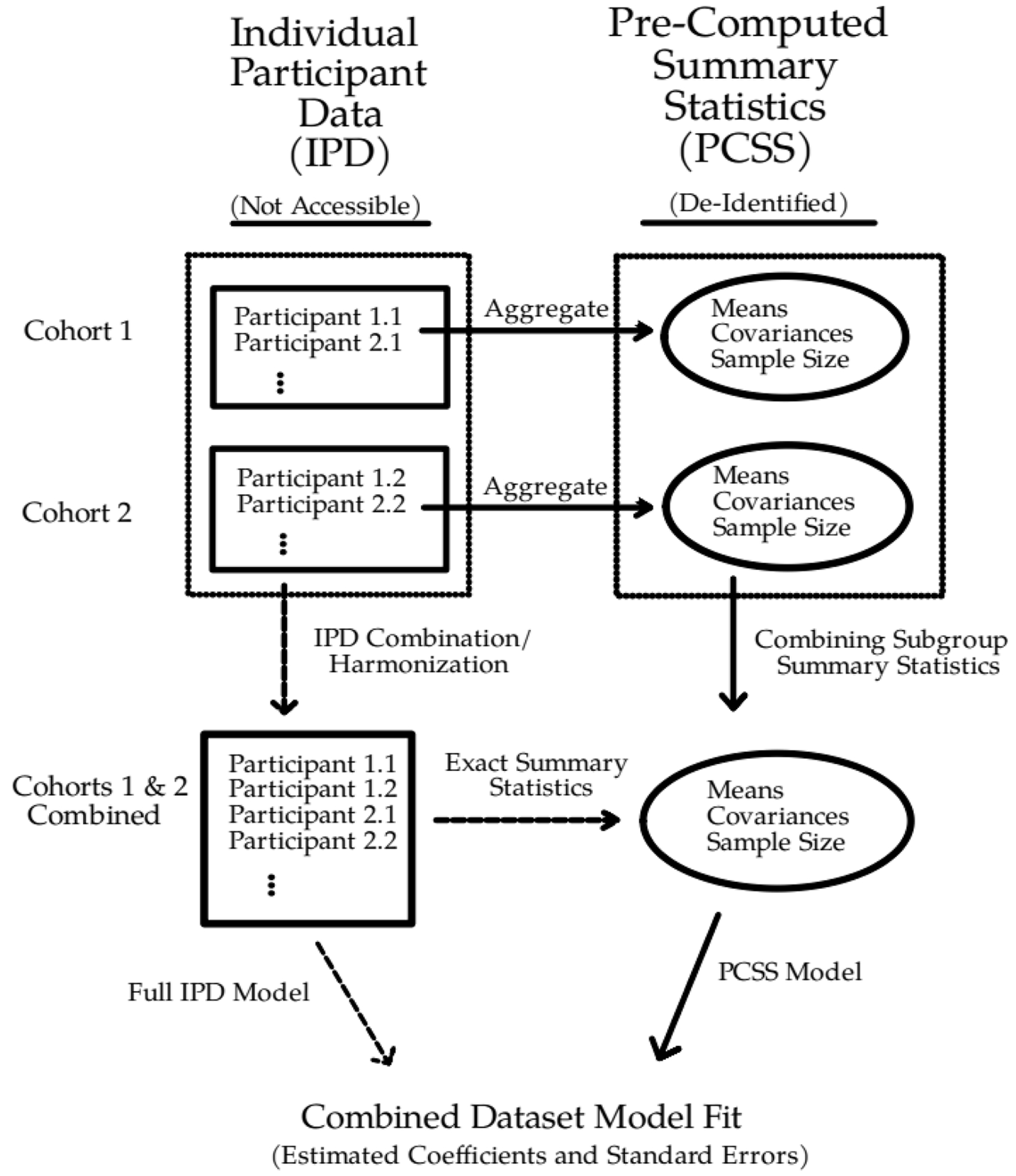
Depiction of the pre-computed summary statistics (PCSS) method using two study cohorts. The PCSS from each individual study can be combined into the same PCSS that would result from a single combined dataset across all cohorts (e.g., IPD from all studies). From these PCSS, we can subsequently compute linear model estimates (point estimates; SEs) for the combined dataset identical to those that would be obtained as if we had IPD for the multi-cohort dataset. Solid arrows demonstrate the summary statistics process, while dashed arrows represent the underlying relationships that allow the exact calculation of a combined dataset model fit. While the figure illustrates the combination of two cohorts for simplicity, the approach works for as many cohorts as PCSS data is available.

With only the pre-computed summary statistics (PCSS) from a study, we can produce the exact ordinary least squares (OLS) model fit (point estimates; standard errors (SEs) and predictions from the standard approach used when IPD data is available) from any outcomes (*y*), predictors (*x*), and other covariates that were summarized [24,25]. In short, this method can calculate an exact OLS model fit of any subset of outcome and predictor variables for which we have averages, covariances, and know the sample size across all the chosen variables. Furthermore, this emerging PCSS-based method can produce exact model estimates for linear combinations of outcome variables (e.g., y_1_ - y_2_), based only on PCSS data for y_1_ and y_2_. In this paper, we expand the method to a multi-cohort setting, mathematically combining PCSS from multiple cohorts to produce the exact set of summary statistics that would result from a single combined dataset of all cohorts. We then demonstrate how to calculate exact linear model fits as if we had IPD from each cohort in a single combined dataset. Fig. 1 provides a high-level overview of the emerging methodology as compared to a standard approach given access to IPD.

### 2.2. Methodological details

Let *k* represent the number of cohorts that will be combined and *n_j_* represent the sample size of the *j^th^ (i* = *1*,…,*k*) cohort. If we had access to IPD on all cohorts and combined the cohort-level IPD into a single, harmonized multi-cohort set of IPD, then in order to investigate the association between a single explanatory variable of interest (*X_1_*) with a single response variable (*Y*), after adjusting for relevant covariates (*X_2_,…,X_m_*), and adjusting for fixed cohort effects (*C_1_,…,C_k_*), we would typically use the following linear model:

(1) $$Y=\beta_{0}+\beta_{1}X_{1}+\ldots +\beta_{m}X_{m}+\gamma_{1}C_{1}+\ldots +\gamma_{k}C_{k}$$

Typically, our focus would be on the point estimate $\hat{\beta_{1}}$ and associated standard error $SE(\hat{\beta_{1}})$, though the results here hold for any $\hat{\beta_{i}}$ or $\hat{\gamma_{j}}$. We can calculate $\hat{\beta_{1}}$ and $SE(\hat{\beta_{1}})$ using the means $\left(\bar{X_{j}}\right)$, samples sizes (*n*), and covariances $\left(Cov\left(X_{i},X_{j}\right)\right)$ using the PCSS (see [24] for full mathematical details). Furthermore, we note that these results also hold for interactions (e.g., *X_1_X_2_*) or polynomial (e.g., $X_{1}^{2}$) terms, as long as the interaction or polynomial term is part of the original (cohort-specific), available PCSS. Similarly, alternative forms of variables (e.g., continuous vs. categorical age), must first be available in the cohort-specific PCCS, but then can be easily used in all downstream model-building. Importantly, we can also perform ANOVA F-tests for model comparison (e.g. to test for inclusion of an interaction term) [27]. Finally, to perform a subgroup (stratified) analysis, PCSS for each strata in each cohort must be available, which can later be combined to reflect the full cohort datasets (see the following section). All PCSS methods used in this paper are integrated into an R software package (pcsstools) published on CRAN [14].

### 2.3. Combining subgroup summary statistics to recover full group summary statistics

To facilitate multi-cohort analysis, we have developed a technique to combine several sets of PCSS from different cohorts into the exact set of PCSS that would result from a single combined set of IPD. Our technique to calculate the covariance matrix of the single combined dataset leverages the decomposition of total deviation (sums of squares) which is the basis for an ANOVA F-test. We now provide an overview of the approach.

Without loss of generality, we will examine any two explanatory variables *X_1_* and *X_2_*. First, we calculate “group” sum of squares for *X_1_* and *X_2_* as: $SSG=\sum_{j=1}^{k}\;n_{j}({\overset{‾}{X}}_{1j}-{\overset{=}{X}}_{1})({\overset{‾}{X}}_{2j}-{\overset{=}{X}}_{2})$, using the cohort-specific $\left({\overset{‾}{X}}_{j}\right)$ and overall $\left({\overset{=}{X}}_{j}\right)$ means. Similarly, we get the error sum of squares $SSE=\sum_{j=1}^{k}\;\sum_{i=1}^{n_{j}}\;\left(X_{1ji}-{\overset{‾}{X}}_{1j}\right)\left(X_{2ji}-{\overset{‾}{X}}_{2j}\right)=\sum_{j=1}^{k}\;\left(n_{j}-1\right)cov\left(X_{1j},X_{2j}\right)$, using the covariances of the two variables in each cohort. These two results allow us to compute the total sum of squares as SSTO=SSG+SSE, which we can scale by total sample size *N* to obtain the sample covariance $\left(Cov\left(X_{1},X_{2}\right)\right)$ for variables *X_1_* and *X_2_* that would result from multi-cohort IPD. The previous result holds for any disease or biomarker response variables. Parallel reasoning can be applied to obtain a variance (e.g. $Cov\left(X_{1},X_{1}\right)$). Once we have the means, sample size, and covariance matrices for all variables, we can apply the approach of the previous section to obtain all linear model point estimates and SEs. In addition, it is also straightforward to produce the means and covariances of indicator variables for cohort membership and include fixed effects for each cohort in the resulting linear models. In summary, this approach illustrates how, when we have PCSS for individual cohorts, we calculate the exact PCSS that would result from multi-cohort IPD, yielding the same OLS point estimates and SEs that would be obtained as if IPD access to all cohorts were available.

### 2.4. Framingham heart study

The Framingham Heart Study is a population-based, multi-cohort, longitudinal study of families in Framingham, Massachusetts. We use four separate cohorts from the Framingham Heart Study: Offspring, Generation 3, Omni I, and Omni II, which each have RBC membrane fatty acid and Trail Making Test measurements. The Omni cohorts were recruited to promote inclusion of other races and ethnicities to the study, and are much more diverse than the Offspring and Generation 3 cohorts which are primarily Caucasian. In order to limit temporal confounding, we limit our sample to individuals who have FA measurements within 5 years of TMT. Given our focus on assessing cognitive impairment in individuals without dementia, we also only included participants between 50 and 70 years old and removed participants with dementia at the time of the Trail-Making Test, resulting in a final combined (multi-cohort) sample size of *N* = 2158. See Table 1 for cohort-level and participant removal sample sizes.

### 2.5. Trail making test

The primary outcome of interest is results from the Trail Making Test, a test of motor skills (Part A) and higher level cognitive function (Part B; e.g., mental flexibility, working memory, task switching) [6,16,20]. To complete the test, participants must draw a trail through a set of randomly placed, numbered dots (part A), then draw a trail through a set of differently labeled dots alternating between numerical and alphabetical orders (part B). In both cases, the participant is timed and instructed to draw the trail as quickly as possible. Beyond the direct measures (Part A or Part B time to completion in minutes), a key outcome obtained from the Trail Making test (and the focus of our analysis) is the difference in times between Part A and Part B: minutes taking part B - minutes taking part A. Taking the difference between parts (B minus A) accounts for individuals’ visuoperceptive ability and provides a measure of executive control [5,6,20]. In short, a larger value of this measure indicates worse executive function and greater cognitive impairment (i.e. the reason a participant takes much longer on part B than on part A is a reduced ability for higher order functioning).

### 2.6. Red blood cell DHA determination

In the FHS cohorts, fatty acids (FAs) were measured from blood drawn after a 10–12 hour fast into an EDTA tube, and RBCs were separated from plasma by centrifugation. The RBC fraction was frozen at −80 °C immediately after collection. RBC fatty acid composition was determined as described previously (OmegaQuant Analytics, Sioux Falls, SD, USA) [12]. Briefly, RBCs were incubated at 100 °C with boron trifluoride-methanol and hexane to generate fatty acid methyl esters that were then analyzed by gas chromatography with flame ionization detection. Individual FAs were identified through comparison with a standard mixture characteristic of RBC and are expressed as the percentage of the total pool of fatty acids. Docosahexaenoic acid (DHA) is the focus of our analysis; the interassay coefficient of variation for DHA was <4 %.

### 2.7. Covariates and APOE status

In the Framingham Heart Study, autosomal SNPs were measured directly using an Affymetrix 500 K chip. Specific SNPs were located as part of previously identified genes using GWAS data from the NHGRI-EBI GWAS Catalog [3]. Using this data, we identified *APOE-ε4* carriership status (1 or 2 copies vs. none) for each participant. Furthermore, we considered the following covariates assessed at the time of FA measurement: age, biological sex, race and ethnicity, education level, marital status, BMI, alcohol consumption (drinks per week), physical exercise (MET-hours per week), use of cholesterol and blood pressure lowering medications, diabetes diagnosis, and cardiovascular disease status.

### 2.8. Statistical analysis

We examined the cross-sectional relationships of TMT times with RBC DHA fraction, and how these associations may differ by *APOE-ε4* status using three separate methods: (a) the novel PCSS-based method described above, (b) using IPD data, and (c) a standard meta-analysis approach [9]. The basis of all models was the prediction of TMT time differences (Part B minus Part A) by DHA levels, while adjusting for all covariates. Additionally, we tested for interaction by *APOE* status (carrier vs. non-carrier), performing an ANOVA F-test for the inclusion of an interaction term in the model. We subsequently fit the same model separately on *APOE-ε4* carriers and non-carriers. For the PCSS-based method, we used the required PCSS data computed separately for each subgroup (*APOE-ε4* carriers and non-carriers) in each cohort (means, covariance matrices, sample size). For the IPD approach, we created a single, multi-cohort, IPD set. For the standard meta-analysis commonly found in the literature, we used fixed-effects meta-analysis on linear model fits computed separately on each cohort’s IPD data. All models were run in R using the lm() function for the IPD analysis and the pcsslm () function from the pcsstools package [14] for the PCSS method. Statistical significance was set to α=0.05 in all cases.

## 3. Results

### 3.1. Sample characteristics

Descriptive statistics for TMT, DHA, *APOE* status, and all covariates are shown in Table 2 – within and across cohorts. By design, the omni cohorts are ethnically/racially diverse, while the Gen 3/Omni 2 cohorts are younger. Otherwise, the demographic, lifestyle behavior and medical history of participants is broadly reflective of U.S. population norms. DHA fraction is fairly low, and after exclusion of individuals with dementia/AD at time of FA measurement, results on the Trail Making Test (Time on B minus Time on A) illustrate little evidence of cognitive impairment overall. Note: We use ‘cognitive impairment’ to reflect this value throughout the rest of the manuscript.

### 3.2. Model results

In Table 3 and Fig. 1 we report the results combined across all four cohorts when exploring the relationship between DHA and cognitive impairment, overall and stratified by *APOE-ε4* status. As shown earlier (Methods), the PCSS method and IPD analyses are mathematically equivalent and, thus, yield identical results. For comparison we also include results from a standard meta-analysis approach. The p-value for the interaction between DHA and *APOE-ε4* status is statistically significant for both the PCSS/IPD approaches (*p* = 0.039) and the standard meta-analysis approach (*p* = 0.047). Stratified estimates are identical for PCSS and IPD, and similar to those obtained in standard meta-analysis. In short, DHA has a statistically significant, negative association with cognitive impairment in *APOE-ε4* carriers (${\hat{\beta }}_{DHA}:-0.13$ min per 1 % increase in RBC DHA; 95 % CI: (−0.20, −0.06)), meaning that higher DHA levels associate with lower levels of cognitive impairment. While the same inverse association is true in non-carriers, (${\hat{\beta }}_{\mathrm{DHA}}:-0.06$; 95 % CI: (−0.11, −0.02)), the strength of association is approximately half.

**Fig. 2.**
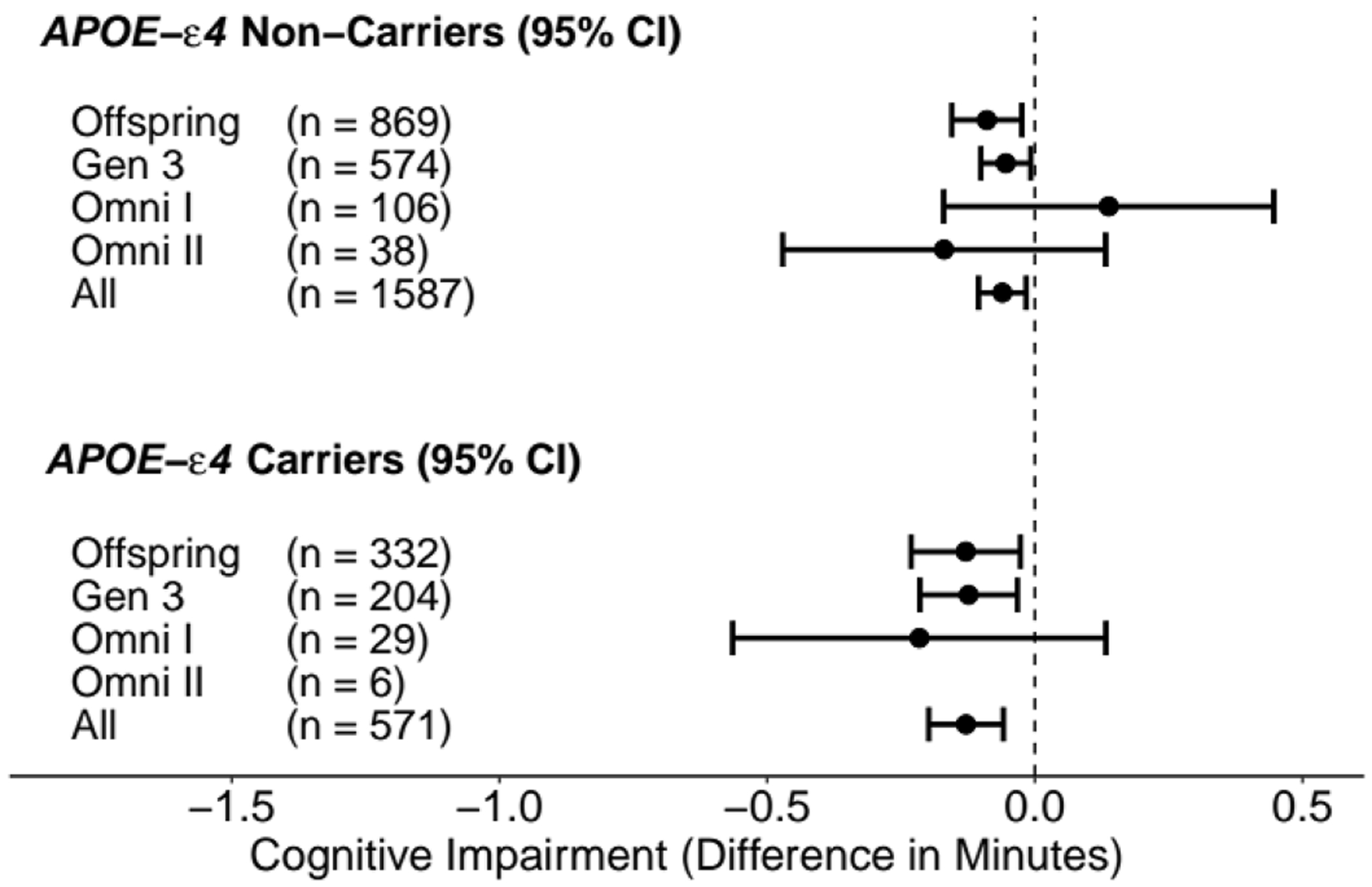
Forest plot of the estimates and 95 % confidence intervals for cognitive impairment per one-percentage point increase in RBC DHA. Estimates are from cross-sectional PCSS models on the summary statistics of datasets stratified by *APOE-ε4* status.

Table 4 contains the results separated by cohort, with Fig. 2 depicting the corresponding results in a forest plot. None of the cohort-specific tests for interaction attain statistical significance. However, all individual cohort stratified estimates are inverse, and stronger in *APOE-ε4* carriers, with the exception of non-carriers in Omni I. However, the confidence interval for Omni I non-carriers includes negative values and is not statistically significant (*p* > 0.05). Table 4 results are shown using only the PCSS approach, though, as noted elsewhere, results are identical to those from IPD.

## 4. Discussion

In this paper, we found RBC DHA fraction to be inversely associated with cognitive impairment among dementia-free participants aged between 50 and 70 in pooled data across four cohorts, and that the association is significantly stronger among *APOE-ε4* carriers than non-carriers. These findings are aligned with the notion that cognitive benefits of DHA-based interventions may be greater in dementia-free *APOE-ε4* carriers [7]. Furthermore, we demonstrated a method for combining data across studies using PCSS that yields results identical to those obtained using IPD. In particular, within this PCSS framework, we were able to perform a stratified analysis (by *APOE-ε4* status), select any subset of variables, and linearly transform (i.e., compute Trails B - Trails A for an outcome), all using only the means, covariance matrices, and samples sizes for *APOE-ε4* carriers and non-carriers in each cohort. Besides establishing further evidence of the link between DHA and cognition (being differential by *APOE-ε4* carriership), we have demonstrated an emerging approach to multi-cohort linear models that circumvents the major roadblock of individual participant data access.

The clinical relevance of our findings relies on the stronger associations observed in *APOE-ε4* carriers. Because of the fundamental role of Apolipoprotein E in cellular lipid transport and metabolism in the brain and periphery, Apolipoprotein E affects DHA metabolism as well. In this regard, *APOE-ε4* carriers display a lower status of DHA, secondary to accelerated liver catabolism of DHA. In addition, *APOE-ε4* produced in astrocytes promotes degradation of the tight junctions and breakdown of the outer leaflet of the blood-brain barrier, therefore hampering brain DHA uptake from the systemic circulation, in particular in young, cognitively healthy *ε4* carriers [7]. This is overall indicative of higher DHA requirements in this specific population subgroup, which can be corrected through dietary supplementation. In contrast, supplementation is regarded to be less effective once the clinical symptomatology appeared, due to accelerated oxidation of DHA [7]. Such notion is consistent with randomized controlled trials examining *APOE-ε4* carriership strata, which reported benefits for DHA and other omega-3 in cognitively healthy participants [18,21], but not in elderly people with memory complaints [1] or with AD/ARDRD [17]. Our findings support future randomized controlled trials focusing recruitment on middle-aged and cognitively healthy *APOE-ε4* carriers.

We note that the PCSS approach is advantageous as it generally will provide more power than standard meta-analysis since the PCSS approach is identical to IPD approaches. Thus, PCSS is both more flexible than standard, literature based meta-analysis (which is further limited by pre-defined, non-harmonized analyses) and at least as powerful. In an age of rapidly growing demands and expectations for data sharing and access, and equally important about data exploitation and privacy, we need to continue to explore innovative ways of enhancing data sharing across cohorts/studies to maximize the number of individuals who can access and explore datasets. PCSS data sharing methods provide such an opportunity, though have been limited in utilization due to the limited number of downstream statistical methods that can leverage the data. Here, we demonstrate how PCSS methods can be used for multi-cohort style analyses. However, to be fully and maximally useful, we must continue to encourage cohort studies and RCTs to publicly share PCSS. This should be imminently doable since PCSS contain no individually identifiable data, and standard IRB permissions include the ability for investigators to publish/release cohort level summary statistics.

While in this study we have only included four cohorts from the Framingham Heart Study, two of which together contributed <200 participants, the PCSS method can take advantage of more and larger cohorts with no limit. Notably, as shown here, the method is limited to applications of ordinary least squares regression with a quantitative outcome. However, ongoing research by our group is exploring nonlinear regression techniques (e.g., logistic regression).

Another limitation of PCSS is the necessity to pre-compute the ‘right’ set of summary statistics. The method shown here relies only on means, covariance matrices and sample sizes. However, stratified analyses and interactions require some forethought to ensure the PCSS from strata and interactions are included. We also note that our analysis of cross-sectional relationships between blood DHA levels and cognition does not suggest causality or temporality: additional studies are needed to provide conclusive evidence of causality. Finally, we focused on the established *APOE* gene, however recent work from our group suggests that there may be more genes beyond *APOE* for which DHA provides differential benefit and is need of further research [2].

In conclusion, our analysis has shown continued evidence of the inverse relationship between blood DHA levels and cognitive outcomes: a relationship that is stronger in *APOE-ε4* carriers. Furthermore, we have demonstrated an emerging methodology for the use of PCSS to enhance data sharing and maximize downstream statistical power for multi-cohort analyses. Further work is needed to continue to expand the methods and encourage PCSS computation and widespread release by cohort studies.

## Figures and Tables

**Table 1:** Sample sizes (*N*) across four Framingham Heart Study cohorts and by data availability, filtering by age, and filtering by time between DHA measurement and Trail-Making Test (TMT).

	Offspring	Generation 3	Omni 1	Omni 2	All
Any available TMT, DHA, *APOE-ε4*, continuous covariates, and without baseline dementia	2222	2181	236	123	4762
After limiting to individuals 50–70y	1389	809	185	53	2436
After excluding individuals with TMT >5 years from DHA measurement	1201	778	135	44	2158

**Table 2:** Descriptive statistics of study participants by Framingham Heart Study cohort and overall. Unless otherwise noted, covariates were measured during the same visit that blood was drawn for red blood cell fatty acid measurement. Values are % or mean ± SD.

	Offspring (*N* = 1201)	Generation 3 (*N* = 778)	Omni 1 (*N* = 135)	Omni 2 (*N* = 44)	All Cohorts (*N* = 2158)
**Demographics**					
Sex, female %	55	51.8	64.4	47.7	54.3
Age at Trail-Making Test, years	61.5 (4.8)	55.0 (4.1)	58.8 (5.4)	57.4 (5.5)	58.9 (5.5)
Race and Ethnicity					
Non-Hispanic White %	98.4	99.1	2.2	0	90.6
NH Black %	0.1	0	37.8	27.3	3
NH Asian %	0	0	23	47.7	2.4
NH Other %	0.7	0.8	0.7	4.5	0.8
Hispanic %	0.1	0	36.3	20.5	2.7
Education					
Less than high school %	0.2	0	7.4	0	0.6
Graduate High school %	22	15.4	14.8	4.5	18.8
College degree or more %	77.3	84.4	76.3	90.9	80.1
Marital Status					
Married/Cohabitating %	74	70.6	68.9	84.1	72.7
Never Married %	5.7	9.8	5.9	6.8	7.2
Widowed %	4.7	2.2	8.9	4.5	4.1
Divorced/Separated %	14.9	17.2	16.3	4.5	15.6
BMI, kg/m^2^	28.4 (5.5)	28.9 (6.0)	28.7 (5.3)	26.4 (4.4)	28.6 (5.7)
**Lifestyle**					
Alcohol, Drinks Per Week	5.1 (7.3)	5.5 (7.9)	2.7 (5.1)	2.7 (4.0)	5.0 (7.4)
Exercise, MET-Hours Per Week	268.7 (97.1)	265.4 (113.8)	257.5 (92.6)	258.9 (100.6)	266.6 (103.2)
**Medical Condition**					
Cholesterol Medication %	41	28.3	43	29.5	36.3
Hypertension Medication %	41.7	27.5	45.2	34.1	36.7
Diabetes mellitus Diagnosis %	10.2	10.4	20	15.9	11
Cardiovascular Disease %	9.3	4.2	9.6	4.5	7.4
*APOE-ε4* Carrier %	27.6	26.2	21.5	13.6	26.5
**Fatty Acid Exposure**					
Plasma DHA, % of Total FA	4.8 (1.4)	4.4 (1.3)	5.2 (1.4)	5.1 (1.0)	4.7 (1.4)
**Cognitive assessment**					
Years Fatty Acid Measurement to Trail-Making Test (date TMT - date FA)	0.2 (1.5)	1.6 (1.0)	−0.2 (1.1)	2.4 (1.1)	0.8 (1.5)
Cognitive Impairment (Trails Part B - Trails Part A, Minutes)	0.9 (0.9)	0.7 (0.5)	1.3 (1.3)	0.9 (0.7)	0.8 (0.8)

DHA = docosahexaenoic acid; *APOE* = Apolipoprotein E; NH = Non-Hispanic.

**Table 3:** Results from all cohorts combined estimating the relationships between cognitive impairment and DHA stratified by *APOE-ε4* status. The PCSS method and IPD analyses yield identical results, and are compared with an inverse-variance weighting meta-analysis on the four cohort model results. Estimated coefficients are reported with 95 % confidence intervals in parentheses.

	PCSS Method^1^	Individual Participant Data Method^1^	Inverse Variance Weighting Meta-Analysis Method
P-value from ANOVA F-test for interaction	0.039		0.047
${\hat{\beta }}_{DHA}$ for *APOE-ε4* Carriers (stratified)	−0.13 (−0.20, −0.06)		−0.13 (−0.20, −0.06)
${\hat{\beta }}_{DHA}$ for *APOE-ε4* Non-Carriers (stratified)	−0.06 (−0.11, −0.02)		−0.06 (−0.10, −0.03)

1 These two methods provide equivalent results by design (see Methods section for details).

**Table 4:** Results from individual cohorts estimating the relationships between cognitive impairment and DHA stratified by *APOE-ε4* status. Point estimates are reported with 95 % confidence intervals in parentheses.

	Offspring (*N* = 1201)	Generation 3 (*N* = 778)	Omni 1 (*N* = 135)	Omni 2 (*N* = 44)
P-value from ANOVA F-test for interaction	0.21	0.12	0.99	0.95
${\hat{\beta }}_{DHA}$ for *APOE-ε4* Carriers (stratified)	−0.129 (−0.231, −0.027)	−0.124 (−0.215, −0.032)	−0.216 (−0.564, 0.133)	Insufficient Sample Size (*n* = 6)
${\hat{\beta }}_{DHA}$ for *APOE-ε4* Non-Carriers (stratified)	−0.090 (−0.155, −0.024)	−0.054 (−0.101, −0.008)	0.138 (−0.17, 0.446)	−0.169 (−0.471, 0.132)

## Data Availability

The data/analyses presented in the current publication are based on the use of Framingham Study data downloaded from the dbGAP web site (phs000007.v35.p16; https://www.ncbi.nlm.nih.gov/projects/gap/cgi-bin/study.cgi?study_id=phs000007.v35.p16), where qualified researchers can apply for authorization to access.
